# Supplementary material for: The Impact of Health and Social Services on the Quality of Life in Families of Adults with Autism Spectrum Disorder (ASD): A Focus Group Study
Source: Brain Sci. 2022 Jan 28;12(2):177. doi: 10.3390/brainsci12020177 (PMC8869802; doi:10.3390/brainsci12020177)
Supplement: Supplementary file 1 [file brainsci-12-00177-s001.zip › Supplementary item 1 Table S1.pdf]

Table S1. Characteristics of the subjects included in the LLIP project.

subject	sex	Diagnosis	support needed	kind of family	informant	sibling (B/S)	sibling (n)	sibling (age)	parent (age)	parent (employment mother)	parent (employment father)	family income perception vs average country income	Family income Self-perception	% family income for special support
2	F	ASD	Level 3	two-parent family	Mother	S	1	-	-	Employed full-time	Self-employed full-time	Above average	Well off	None
3	M	ASD	Level 3	two-parent family	Mother	S	1	22	59	Self-employed full-time	Self-employed full-time	Average	Doing okay	26% to 50%
4	F	ASD	Level 3	two-parent family	Father	B	1							
5	M	ASD	Level 3	two-parent family	Father	B	1	24	58	Self-employed full-time	Self-employed full-time	Above average	Doing okay	26% to 50%
6	M	ASD	Level 3	one-parent family	Mother	B	2	20, 25	48	Employed part-time	-	Well below average	Struggling	None
8	M	ASD	Level 3	two-parent family	Father	B	1	23	56	Employed full-time	Self-employed full-time	Above average	Managing well with some extra	26% to 50%
10	M	ASD	Level 3	two-parent family	Mother	S	1	26	60	Employed part-time	/	Well above average	Well off	26% to 50%
11	M	ASD	Level 3	two-parent family	Father	S	1	26	60	/	Self-employed full-time	Well above average	Well off	26% to 50%
12	M	ASD	Level 3	two-parent family	Father	S	2	28,33	49	/	Employed full-time	Average	Managing well with some extra	None
