# Supplementary material for: The Impact of Health and Social Services on the Quality of Life in Families of Adults with Autism Spectrum Disorder (ASD): A Focus Group Study
Source: Brain Sci. 2022 Jan 28;12(2):177. doi: 10.3390/brainsci12020177 (PMC8869802; doi:10.3390/brainsci12020177)
Supplement: Supplementary file 1 [file brainsci-12-00177-s001.zip › Supplementary item 2 - Service Survey Questionnaire.pdf]

Supplementary material item S2. Service Survey Questionnaire

A. What are the positive aspects of your Health Local Unit (HLU)? Strike the corresponding option/options.

1. Staff competence and efficiency
2. Reduced waiting times
3. Clear communication
4. Offering services
5. Good service organization
6. Ease of access to services
7. Involvement and participation in your child's decisions
8. Other (specify):

____
____
____

B. What are the downsides of your ASL? Strike the corresponding option/options.

1. Poorly prepared and helpful staff
2. Prolonged waiting times
3. Unclear communication
4. Poor service offering
5. Service disorganization
6. Difficulty accessing services
7. Poor involvement and participation in decisions about your child
8. Other (specify):

____
____
____

C. What are the reasons why you get in touch with the ASL services? Strike the corresponding option/options.

1. Certifications/bureaucratic aspects

2. Clinical take-up

3. Health and Social Inclusion Projects

4. Other (specify):

____
____
____

D. On a quantitative level, has the relationship with your ASL been constant or has it varied over time? Strike the corresponding option.

1. It has decreased

2. It has been constant

3. Increased

4. Other (specify):

____
____
____

E. On a qualitative level, has the relationship with your ASL been constant or has it varied over time? Strike the corresponding option.

1. It's got worse

2. It has been constant

3. Has improved

4. Other (specify):

____
____
____

F. Are there any services of your ASL that you would like to see implemented/improved? Strike the corresponding option.

1. Yes

2. No.

3. Non so

G. If so, which ones?

- Day/Semi-residential Centres

Relief services

- Autonomous Living Projects/Co-housing/Protected Apartments

- Continuous care/home service

- Family support

- School-work transition paths

- Job entry paths

- Unique hospital pathways for autism

H. Please describe how you would like to see the services you have indicated or others not listed implemented/improved:

____
____
____

____
____
____

I. In general, what vote would give to your experience with the service of your ASL in the last **12 months**? Strike the chosen option, where 1 stands for "bad experience" and 10 stands for "great experience".

1	2	3	4	5	6	7	8	9	10

J. How would you define the **quality** of your relationship with the relevant municipal figures?

1. Bad
2. Poor
3. Sufficient
4. Good
5. Great
6. Other (specify):

____
____
____

K. What are the reasons why you get in touch with the services of your Town Hall? Strike the corresponding option/options.

1. Welfare benefits (e.g. indirect assistance, economic contributions, educational assistance)
2. Recreational and sports projects and workshops
3. Health and Social Inclusion Projects
4. Summer Stays
5. Other (specify):

____
____
____

L. In general, what is the rating of the experience with the service of your town hall in the last **12 months?** Strike the chosen option, where 1 stands for "nothing" and 10 stands for "very much".

1	2	3	4	5	6	7	8	9	10
